# Commencing and Persisting With a Web-Based Cognitive Behavioral Intervention for Insomnia: A Qualitative Study of Treatment Completers

**DOI:** 10.2196/jmir.5639

**Published:** 2017-02-10

**Authors:** Charles Chan, Stacey West, Nick Glozier

**Affiliations:** ^1^ Brain and Mind Centre University of Sydney Camperdown Australia

**Keywords:** adherence, persistence, eHealth, online intervention, Web-based intervention, motivations, barriers, insomnia, depression, men

## Abstract

**Background:**

Computerized cognitive behavioral therapy for insomnia (CCBT-I) has a growing evidence base as a stand-alone intervention, but it is less clear what factors may limit its acceptability and feasibility when combined with clinical care.

**Objective:**

The purpose of this study was to explore barriers and facilitators to use of an adjunctive CCBT-I program among depressed patients in a psychiatric clinic by using both quantitative and qualitative approaches.

**Methods:**

We conducted the qualitative component of the study using face-to-face or telephone interviews with participants who had enrolled in a clinical trial of a CCBT-I program as an adjunctive treatment in a psychiatric clinical setting. In line with the grounded theory approach, we used a semistructured interview guide with new thematic questions being formulated during the transcription and data analysis, as well as being added to the interview schedule. A range of open and closed questions addressing user experience were asked of all study participants who completed the 12-week trial in an online survey.

**Results:**

Three themes emerged from the interviews and open questions, consistent with nonadjunctive CCBT-I implementation. Identification with the adjunctive intervention’s target symptom of insomnia and the clinical setting were seen as key reasons to engage initially. Persistence was related to factors to do with the program, its structure, and its content, rather than any nonclinical factors. The survey results showed that only the key active behavioral intervention, sleep restriction, was rated as a major problem by more than 15% of the sample. In this clinical setting, the support of the clinician in completing the unsupported program was highlighted, as was the need for the program and clinical treatment to be coordinated.

**Conclusions:**

The use of a normally unsupported CCBT-I program as an adjunctive treatment can be aided by the clinician’s approach. A key behavioral component of the intervention, specific to insomnia treatment, was identified as a major problem for persistence. As such, clinicians need to be aware of when such components are delivered in the program and coordinate their care accordingly, if the use of the program is to be optimized.

**ClinicalTrial:**

Australian and New Zealand Clinical Trials Registry ACTRN12612000985886; https://www.anzctr.org.au/Trial/Registration/TrialReview.aspx?id=362875&isReview=true (Archived by WebCite at http://www.webcitation.org/6njjhl42X)

## Introduction

Depression is one of the most common psychiatric disorders in older adulthood and accounts for much of today’s health care burden placed on medical professionals [1].

The recent development of eHealth technology has led to many efficacious interventions for depression being provided consistently to large numbers of people [2]. Computer-delivered therapies (often based on cognitive behavioral therapy [CBT] principles and thus termed computerized CBT [CCBT]) are more readily accessed by individuals who may regard formal mental health services as stigmatizing [3]. Typically, men are among the non-help-seeking group who may benefit from CCBT, as it is less costly and confrontational than face-to-face therapy, while still easily accessible via the growing utility of the Internet and smartphones [4]. When used correctly, CCBT can produce treatment effects similar to those observed in face-to-face treatment; however, those that have therapist interaction are shown to be more effective [2]. In their study, So et al [5] reported limited long-term gains from CCBT, along with an increased rate of participant withdrawal. This effect may be remedied by clinician program support [5]. A growing body of evidence shows that clinician-assisted CCBT results in significant improvements in patients with depressive [2,6] and anxiety disorders [2,7], with results comparable with those obtained from face-to-face treatment.

With rising health care costs, such CCBT programs have been proposed in clinical guidelines to help reduce the cost burden in primary and secondary health care. In 2006, the National Institute for Clinical Excellence [8] (now the National Institute for Health and Care Excellence), United Kingdom, included CCBT as first-step intervention in stepped-care treatment models. In these models, lower-cost interventions are tried first, with more-intensive and -costly interventions reserved for those insufficiently helped by the initial intervention [9]. Such models have proved to be cost effective for depression [10].

In reality, many such CCBT programs are used as adjunctive treatments to face-to-face or even telephone-delivered therapies, rather than with clinicians acting as adjuncts to the program. There is evidence that such adjunctive eHealth programs can add to the effectiveness of existing therapy. For example, Danaher et al [11] showed that combining a Web-based tobacco cessation intervention with using nicotine lozenges encouraged long-term tobacco and smokeless tobacco abstinence. There have been similar observations in other medical conditions, such as enhancing diabetes self-management as an adjunct to regular clinical support, and enhanced results of Internet toilet training for children with encopresis [12].

Given high levels of multimorbidity, and the heterogeneous nature of many psychiatric disorders, a further avenue in improving outcomes is the use of eHealth treatments for comorbidities or symptomatology of depression as adjunctive processes to face-to-face treatments. Insomnia is a frequently reported sleep disturbance in older adults with depression [1], and recent studies have shown that addressing insomnia by using CBT for insomnia can improve depression outcomes [13].

There are now efficacious computerized CBT for insomnia (CCBT-I) interventions [14], with evidence that they have some additional benefit on mood symptoms [15]. A recent study by Christensen et al [16] found that SHUTi significantly lowered depression symptoms on the Patient Health Questionnaire at 6 weeks and 6 months compared with HealthWatch, a general health psychoeducation program.

With the implementation of these adjunctive eHealth treatments into routine clinical practice, emphasis must be placed on process and other logistical concerns. It is less clear what factors may limit acceptability and feasibility of the effects seen in the Internet-only CCBT-I trials when used in clinical practice. Of particular interest are the ways in which patients are able to adhere to intervention techniques provided by the program with respect to timing, dose, and frequency (adherence), and the duration of time in which they are able to actively engage with therapy from initiation until discontinuation (persistence) [17]. Qualitative studies have implicated personal, design, and environmental factors associated with adherence and persistence to CCBT for both depression [18] and insomnia [19]. Adherence to Web-based programs [18] is related to outcome for depression. Further to this, clinicians making a referral or recommendation to a CCBT-I site enhance adherence to the program, suggesting therapy outcomes may relate to clinician interaction [20]. The purpose of this study was to explore the barriers and facilitators to the use of an adjunctive CCBT-I program among depressed patients alongside clinical care.

## Methods

### Study Sample

Participants in the study were primarily residents of New South Wales, Australia, who had enrolled in the Sleep Or Mood Novel Adjunctive therapy (SOMNA) trial, which included a CCBT-I program used as an adjunctive treatment in a psychiatric clinical setting [21]. All participants were English speaking, had an active email address, and currently met the criteria for at least minor depression, defined as screening positive on the Quick Inventory of Depressive Symptomatology [22]. A diagnosis of depression was then confirmed at a clinical interview by a psychiatrist, using *Diagnostic and Statistical Manual of Mental Disorders* (DSM-5) criteria for either a major depressive disorder or dysthymia, the Structured Clinical Interview for DSM-IV [23], and clinically significant insomnia symptoms reported on the Insomnia Severity Index [24]. Due to funding conditions, participants were male and aged 50 years or over. We excluded participants if they (1) had a history of psychosis or hypo/manic episode, (2) had a current substance dependence, or (3) had a score of <24 on the Mini-Mental State Examination, all as determined by the psychiatrist clinical interview; (4) were a rotating shift worker with overnight shifts, or with transmeridian travel >2 hours in the past month, (5) had all the criteria for restless legs syndrome as defined using the Cambridge-Hopkins Restless Legs Syndrome Short Form Questionnaire at screening, or (6) were at high risk for obstructive sleep apnea as defined by the Berlin Questionnaire or had been treated for obstructive sleep apnea. Participants were randomly allocated to either the active intervention of CCBT-I program or to an active control of Internet-delivered sleep health information.

### Clinical Setting

The participants were treated at a university psychiatric clinic staffed by consultant, final-year trainee psychiatrists, or both. Standard clinical care for treatment of depressive symptoms was delivered as considered clinically appropriate by the psychiatrist in conjunction with the *beyondblue* clinical guidelines [25]. This included watchful waiting, psychotherapy referral, or medication use under standard collaborative care with a general practitioner. As part of their standard treatment, participants were required to undertake a semistructured clinical assessment with a psychiatrist, whereupon a treatment plan was developed. This assessment included administration of standard clinical outcome measures, including the Hamilton Rating Scale for Depression, as well as completion of a full medical history. After 2 weeks, participants were required to undergo a clinical review and management appointment with the psychiatrist, in accordance with their treatment plan. This was reviewed at the first follow-up appointment at week 3. Over the subsequent 10 weeks, participants had further clinical review and management as clinically required. This management could also include the psychiatrist undertaking time-limited or supported therapies (although not focused on sleep problems), and referral to or liaison with psychologists, exercise physiologists, other allied health staff, and primary care. Standard clinical instruments were administered to monitor depressive symptoms over this period. If any changes to medication dosage were required, this would be discussed between the patient and psychiatrist or referring doctor.

#### CCBT-I Intervention: SHUTi

SHUTi (Sleep Health Using the Internet, BeHealth Solutions, LLC, Charlottesville, VA, USA) is a 9-week CCBT-I intervention. The program contains elements of psychoeducation, activity scheduling, thought challenging, problem solving, and interpersonal therapy. A major component of the program is sleep restriction, a standard behavioral strategy involving restricting the participant’s time-in-bed (sleep window) to match their average self-reported total sleep duration. The sleep window is then titrated weekly based on sleep efficiency (the proportion of time-in-bed spent asleep), in order to arrive at the participant’s core sleep requirement. Decreasing the opportunity to sleep over successive nights is postulated to build homeostatic sleep pressure, stabilize circadian control of sleep and wakefulness, and dampen presleep cognitive and physiological arousal.

**Figure 1:**
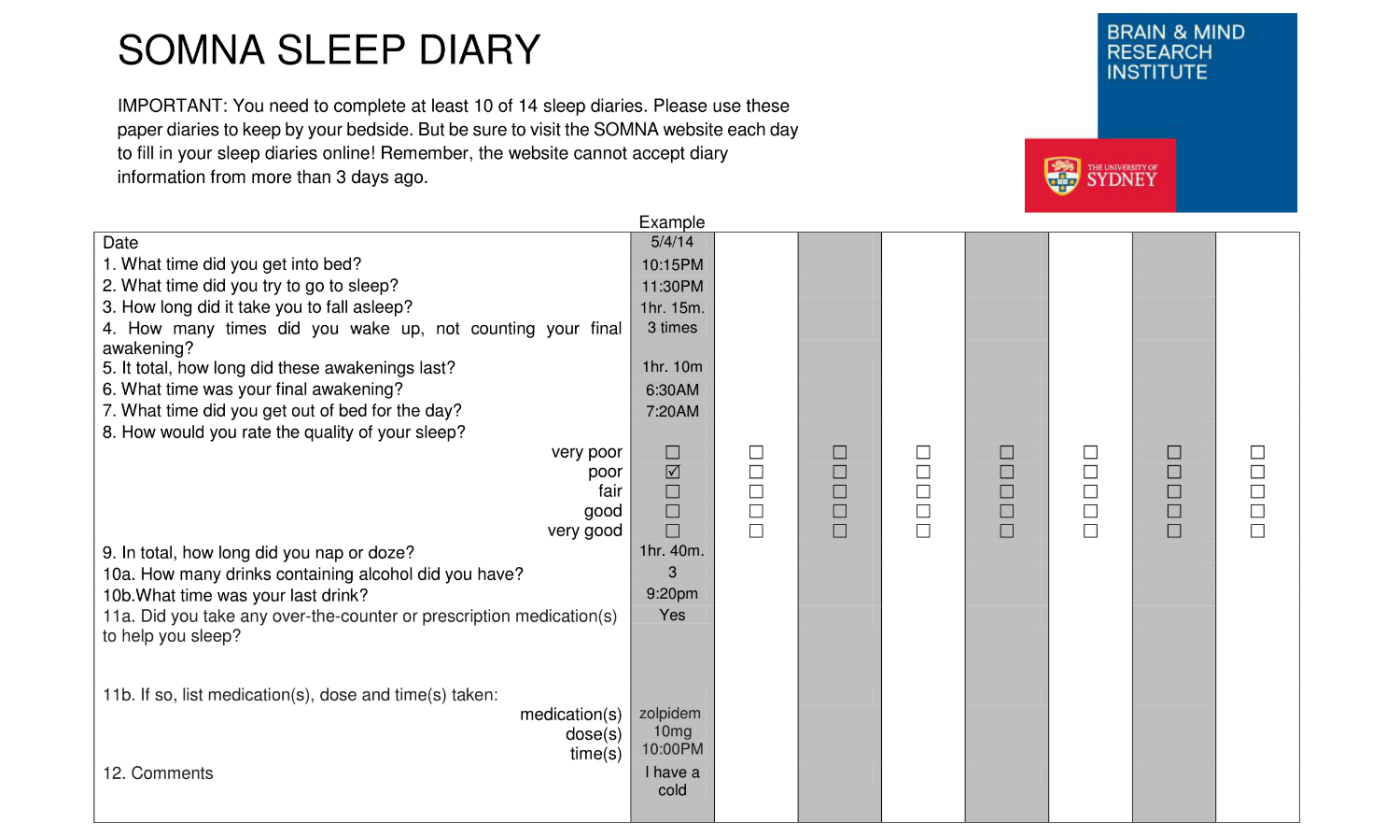
Sample of the Sleep Or Mood Novel Adjunctive therapy (SOMNA) trial sleep diary.

The SHUTi program is fully automated with a new module opening each week after the last has been completed. It is organized into 6 weekly sessions, providing an overview in week 1, and focusing on sleep behavior in weeks 2 and 3, sleep thoughts in week 4, psychoeducation in week 5, and problem prevention in week 6. The participants were able to use the program over the 9 weeks to the end of the trial and then for a further 6 months if required. During the trial, participants received a reminder email 3-4 days after the module was opened or sleep diaries were due. Figure 1 is a sample of the sleep diary. The intervention was delivered alongside the clinical care, but the participants were repeatedly made aware that they could not communicate the components of their Internet program to the clinician to prevent loss of blinding.

#### Online Survey and Analysis

All participants who successfully completed the SOMNA trial were invited to take part in an online program evaluation survey immediately postintervention. The survey contained the Internet Intervention Adherence Questionnaire [26], together with some specific a priori questions relating to CCBT-I. This rates people’s experiences and problems in adhering to the program on a 3-point scale. We grouped these into “major problem,” “minor problem,” and “no problem” and report results as n (%) for each. Within the survey, participants were presented with 2 open-ended items regarding their satisfaction with the program and their rating of the effectiveness of the SHUTi intervention in meeting their personal needs, as well as things such as the ability to offer constructive feedback about the overall user experience and accessibility of the program.

#### Semistructured Interviews and Analysis

Participants were recruited from those who had completed the final 6-month follow-up assessment (including the online survey above) of the SOMNA trial active arm by a research assistant independent of the SOMNA trial. We invited potential participants to the study by email and selected all interested respondents to participate. Following provision of a participant information statement and an overview of the study conduct, we asked potential participants to confirm participation as per written consent form, which stipulated that interviews would be audio recorded using an electronic recording device. We conducted recruitment and interviews in an iterative fashion, with transcripts being reviewed and responses collated, and continued until we reached theoretical saturation (where no new insights emerged from the data for 3 consecutive interviews). Saturation was achieved at the point of preliminary thematic analysis when participants did not report to the interviewer any information that constituted a discrete theme. This occurred after 7 interviews, and was confirmed by the interviewer at interview 10. Given the method and population targeted for recruitment, it was unlikely that subsequent interviews would produce novel or significantly disparate themes on analysis. We offered participants an interview time at their convenience, and we conducted interviews either face-to-face in a clinical setting or via telephone. Travel reimbursement was made available to face-to-face interviewees, although no participants required this.

Using a grounded theory approach, the chief investigator (NG) of the SOMNA trial developed a semistructured interview guide, allowing for new questions to be formulated and added to the interview schedule throughout data collection. Consequently, the interview schedule evolved over the course of the research project. The final interview guide included 10 primary items of interest: (1) overall experience of participation in the combined Internet and clinical interventions, (2) motivation to participate in SHUTi, (3) initial expectations about the Web-based intervention, (4) how expectations were met, (5) how expectations were not met, (6) motivation to continue with Web-based modules and diaries, (7) challenges while doing the modules, (8) experience of clinical treatment and Web-based program adjunctive delivery, (9) suggested improvements to SHUTi for general use, and (10) other reflections or comments on program user experience. Interviews were between 26 and 42 minutes in duration, with all interviews revolving around 1 key question: “What motivated you to persist with the [Web-based] SHUTi program?”

On completion of the interviews, an external agent transcribed the audio files, and the interviewer cross-checked the transcripts against the audio recordings. In conjunction with observational field notes, the chief investigator (NG) and coinvestigator (CC) then collaboratively coded the interview transcripts for recurrent response features. This was followed by focused thematic analysis, where we defined and pursued a central set of themes based on the prevalence and frequency of codes identified by the primary researcher. Conceptual memos were written from the focused themes to help develop an understanding of the themes and how these related to the data. We used no coding or analysis software for this analysis. We used method triangulation to check consistency.

### Ethics Approval and Registration

We obtained written informed consent from all participants engaged with the SOMNA trial. Ethics approval for the SOMNA research trial and this qualitative substudy was obtained from the University of Sydney Human Research Ethics Committee, Sydney, Australia. The trial was registered on the Australian and New Zealand Clinical Trial Registry (ACTRN12612000985886).

## Results

### Sample

A total of 39 of the 40 (98%) eligible participants who commenced the SHUTi program and remained in the trial (regardless of their level of adherence to the modules) completed the survey. Participants were male and had an average age of 59 years. Of the 39 completers, 29 (74%) were married, 36 (92%) had completed post high school qualifications, 33 (85%) met DSM-5 criteria for a major depressive disorder, and 13 (33%) had self-reported cognitive complaints. Due to the anonymous nature of the interview protocol, we did not ascertain demographic data for the qualitative study participants.

### Online Survey

Only one aspect of the program was reported as a major problem, and thus potentially a barrier to adherence, by more than 20% of the participants (Table 1). Difficulty with engaging with the sleep restriction component of the program was reported as a major problem by 12 of 39 respondents (31%). Approximately 1 in 9 participants reported major problems with external factors such as family issues (13%), work issues (10%), and no time (15%), and 13% of participants reported their sleep efficiency not improving. More than half (26/39, 67%) of participants found that CCBT-I improved the problem for which they sought help (“mostly,” 11/39, 28%; “very,” 15/39, 39%). Additionally, some participants (15/39, 39%) reported the program helped them feel prepared to handle this problem in the future (“most,” 8/39, 21%; “very,” 7/39, 18%).

**Table 1:** Self-report problems encountered during the Web-based SHUTi program (n=39).

Problems	Not a problem, n (%)	A little problem, n (%)	A major problem, n (%)	Not applicable, n (%)
Daily requirements were too much for me to do	34 (87)	2 (5)	3 (8)	0
Diaries were too long to complete	34 (87)	4 (10)	1 (3)	0
I had family issues	20 (51)	5 (13)	5 (13)	9 (23)
My family was on the computer	34 (87)	0	0	5 (13)
I had work issues	24 (62)	2 (5)	4 (10)	9 (23)
I forgot to enter data	21 (54)	11 (28)	3 (8)	4 (10)
I had a bad Internet connection	31 (80)	2 (5)	1 (3)	5 (13)
I had a slow Internet connection	29 (74)	3 (8)	1 (3)	6 (15)
I had no time	23 (59)	8 (21)	6 (15)	2 (5)
The screen was hard to read	37 (95)	2 (5)	0	0
Homework amount was too much	25 (64)	9 (23)	0	5 (13)
Homework was difficult	32 (82)	2 (5)	0	5 (13)
Sleep efficiency was not improving	14 (36)	16 (41)	5 (13)	4 (10)
Sleep restriction requirements were difficult	10 (26)	14 (36)	12 (31)	3 (8)
Too many sleep rules to follow	21 (54)	13 (33)	2 (5)	3 (8)
My spouse did not like the program	28 (72)	1 (3)	0	10 (26)
Too much text	28 (72)	10 (26)	1 (3)	0
Website had a bad connection	33 (85)	2 (5)	0	4 (10)
Website was difficult to understand	35 (90)	4 (10)	0	0
Website was hard to navigate	37 (95)	1 (3)	1 (3)	0
Website did not seem useful	32 (82)	2 (5)	5 (13)	0
Website was too long to do	29 (74)	9 (23)	1 (3)	0

### Qualitative Study

We conducted a total of 4 telephone and 6 face-to-face interviews between September 2014 and February 2015.

Three main themes emerged from the qualitative interviews: (1) reasons to commence, and expectations prior to commencing the program related to intrinsic motivators, (2) motivators and barriers related to the program structure and content to continue with the program, and (3) clinical support. The content extracted from the face-to-face interviews regarding the motivators and barriers to continue with the program was triangulated with that collected from the self-report.

#### Reasons for Initial Engagement

##### Identification With the Adjunctive Component

Most participants reported they could identify themselves on the advertisement, which displayed the study inclusion criteria (men, age ≥50 years, with insomnia and stress). The advertisement was presented in various media forms (eg, magazines, online, poster) and was viewed by the participant prior to any involvement or enrollment with the SOMNA clinic. Participants reported being led by advertising material to approach the clinic in the first place, despite many already being in treatment for depression. Some stated they had ignored their insomnia for some time and the advertisement acted as a prompt to do something about it. Linked expectation included the hope that the program would improve their sleep. Some added they had hoped the program could also improve their mood and energy level (interviews 3 and 5). Surprisingly, some participants stated they had no real expectation of the program. Participant 7 said he had tried a few different methods but had not really found much success. He said he participated to “give it a go.” Most participants reported an intrinsic motivation to improve their health holistically. They understood that there was no “quick fix” for their insomnia, and they wanted to understand what the maintaining factors for chronic insomnia were.

##### Clinic Setting in a University

A total of 2 participants stated that they were interested in being involved in clinical research.

I try to be evidence based in my professional work, so I thought, well I will probably get a higher standard of care if I am on a trial than if I just go to some clinic or private practice.

Interview 6

This was echoed in perceptions that this setting might help where previous approaches had failed—for example, reporting having tried various types of remedies with little benefit. In some cases, this had led to some fears of entering the program, that “nothing works,” and even “anticipating failure” (interview 6).

#### Motivators to Persist With the Program

##### Design and Structure of the Program

Most participants reported they found the program easy to use and the contents easy to follow. Participant 1 said

I enjoy the visual representation…it feels good looking at the graphs…I look forward to putting them in to see how I went with getting my sleep efficiency and then felt good when I achieved that efficiency.

Participant 2 agreed that the program fit well with the “visual person.” Participant 5 said that it suited his goal-directed personality and he found it rewarding to see the results.

##### Cognitive Component Through the Program Content

Most participants reported benefits from psychoeducation on sleep patterns, including understanding different factors that can influence sleep, developing good habits, and challenging cognitive misconceptions about sleep behaviors. Participants reported making “cognitive changes” and letting go of rigid reinforcing beliefs.

I learned from the program that it is okay to have minor setbacks…I was able to move on and try again the following night.

##### Behavioral Component: Reinforcing Concepts Throughout the Program and Forming Habits

Participants reported that reinforcement of concepts throughout the modules made it easier to remember them when faced with challenges and insomnia. Most participants found completing a daily sleep diary helpful in making them aware of their sleeping pattern, and also allowing them to go back and review the patterns. This actual behavior and the feedback from the program became self-reinforcing during the program.

It becomes a habit for you so it was easier and that became the motivation, it was just something that you got up and did.

Interview 5

Some identified the behavioral focus early on in creating good habits.

The only way that it is going to be of any benefit to me is if I am regimented with, and do exactly, what they have asked me to do and just have faith in what was said, even though I am thinking how do I go to bed at one o’clock?

Interview 1

I think if in one area of your life you can become disciplined and regimented with it, that can help you to get disciplined in other areas of your life so it can have a flow-on effect.

##### Impersonal and Immediately Accessible Nature of the Internet

Some of the participants preferred the impersonal nature of using the Web over going through the therapy with a psychologist.

The impersonal nature of using the Web was helpful for me. If I had to sit with a psychologist and go through the cores, the experience would be vastly different.

The convenience and constant availability were also identified as the practical benefits of the Internet program in the postintervention feedback.

#### Barriers to Persisting

##### Design and Structure of the Program

On the self-reports, 2 participants suggested using newer graphics and videos. One participant, who worked in a graphic design background, stated “It could look more modern, as it is very conservative, but perhaps the current look is targeted to my demographic or older.” It was also suggested that the program could provide videos produced in Australia (the current videos are produced in the United States).

One participant found 1 of the modules to be too long. He said that “there was too much readings and going to next pages and back again. This would have been more beneficial in shorter modules.”

Some participants reported difficulties in completing the sleep diaries. One of them stated “the methodology to manually log sleep is crude and unreliable. The watch or other devices are more accurate method and should be integrated with the log.” In the self-reports, 3 participants commented that there should be more flexibility with making entries in the sleep diaries. One of them suggested a daily sleep chart question on daily health and whether it impacted on sleep. Some also reported a sense of failure if they did not complete the modules and diaries.

##### Technical Issues

Several recommendations were made regarding the technical aspects of the program. There were 2 participants who commented that the program should have increased compatibility with Apple iPad devices. One of them stated

Some sections within each of the cores would not appear on my iPad. The page would have the border etc but would be blank inside, next page and previous page would be fine. This technical issue needs fixing.

Other suggestions include

A facility to retrieve or change one’s password is imperative. In addition to using dropdowns you should allow people to enter the information directly, such as typing minutes. When the user logs out the webpage is dimmed, which is not a good way to...

A way to jump to any particular part/page directly or with a minimum key strokes.

More printouts and more written information.

Provide examples or interpretations of the intent behind various questions: eg, “how long did it take you to fall asleep?”—Is this during the sleep window (as the context suggests) or generally?

Graphs only depicted in 1-week lots—it would be great to be able to nominate a time period or range of dates and see it all on one graph (therefore see actual trend line of improvement). More personal stories in video format please, need to see in time.

Participant 10 had no access to a computer for a period during the treatment program so he could not respond to reminders, surveys, and emails.

##### Behavioral Component: Difficulties Adhering to Sleep Window

A key behavioral component of CBT for insomnia is sleep consolidation. This process involves using the data derived from people’s sleep diaries to identify the optimum period and duration for their individual sleep. Counterintuitively, this usually leads to a period of sleep restriction. Most participants found it challenging to stick with the sleep window, in particular finding it difficult to stay up until the designated sleep time. For example: “I would sit there and I was in no-man’s-land.” Some were concerned about the effects of the fatigue due to the initial small sleep window on their ability to function and work the following day. Some suggested users should be advised to identify a window of time for their period of sleep deprivation. It was also difficult to stay awake well after the bedtime of their partners, something that integration with the clinical aspects may have enhanced.

#### The Use of CCBT-I in a Clinical Setting

Due to the nature of the double-blind randomized trial, clinicians were unaware whether a participant was allocated to the active computer program intervention or the generic psychoeducation program. It was therefore not possible to integrate the clinical care fully into the computer program intervention in this study.

Nevertheless, most participants found the comprehensive psychiatric assessment and management helpful both for themselves and in persisting with the program: “I would not have completed the program without the clinical support” (interview 3). Conversely, 1 participant perceived the clinical setting as a potential barrier because of either the stigma associated with psychiatry (interview 5) or the logistical difficulties of attending a clinic during working hours.

Two participants (2 and 10) commented that the clinical and computer program interventions and advice seemed disjointed at times. One commented that there may have been a disconnect between the computer results and the clinical assessment, and the clinicians could have been more in tune to the participants’ input in the Web-based program in regard to sleeping patterns (interview 2).

## Discussion

This study aimed to identify the factors influencing adherence to adjunctive CCBT-I in a clinical setting delivering treatment for depression and insomnia. Three themes emerged from the interviews and self-report questionnaires: (1) reasons to commence, and expectations prior to commencing the program, which related to intrinsic motivators, (2) motivators and barriers to continue with the program in relation to the program structure and content, and (3) the interaction of the program with clinical management.

### Reasons to Participate in and Commence the Program

Most of the participants who underwent qualitative interviews about the SOMNA trial stated they could identify themselves in the advertisement. Some had previously been unsuccessfully treated for insomnia and depression. Some reported an intrinsic motivation to better their health holistically, which helped this group of participants persist with the program. The belief in recovery and helpfulness of the program as suggested by Barazzone et al [27] appeared to foster establishment of a therapeutic relationship with the program.

Some participants were attracted to joining the program, as it was conducted within a clinical trial setting, despite seemingly having little expectation or incentive. The reasons, of perceived better quality of care in a university trial setting, were slightly different from the more altruistic reasons for engaging in a trial, that it would benefit others, reported by Donkin and Glozier [18].

### Motivators and Barriers to Continue With the Program

#### Program Structure

It appeared that the program structure of SHUTi had facilitated the participants’ adhering to the Web-based intervention. A majority of participants reported they derived clinical improvements from SHUTi in having a structure to follow. Evidence showed that improving therapeutic relationships would foster a working alliance, which might be associated with proactive information seeking and adherence to eHealth programs [28]. Our participants reported that visual representations had helped them to understand the concept of sleep efficiency and how to improve it. This is in line with the findings that information visualization could be an effective supportive application to improve people’s health literacy [29].

#### Cognitive Component: Psychoeducation Through the Program Content

In our study, psychoeducation (on insomnia) appeared to be regarded well by the participants. Many of the participants reported gaining more knowledge during the program. This was particularly true for those who had ignored their insomnia as a problem for a long time and had little or no previous interventions for their insomnia. Psychoeducation corrected the participants’ misconceptions about sleep behaviors. The results from the survey showed that more than half of the participants found the website improved their insomnia and helped them feel prepared to handle symptoms of insomnia in the future.

#### Behavioral Component: Reinforcing Concepts Throughout the Program and Forming Habits

The visual presentation in SHUTi, such as the graphic interpretations of sleep efficiency, was cited as particularly beneficial for those who had a goal-directed personality. The videos appeared to help participants to relate to others with similar sleep problems. Key concepts were reinforced throughout the modules, and this helped the participants in forming habits, making it easier to persist with the program.

The data entry and self-evaluation of sleep efficiency enabled the participants to review their sleep patterns and receive tailored feedback on their progress. The Web-based program also allowed the participants to learn and practice at their own pace. In this way, these appear to provide feedback, responsiveness, and flexibility, as identified by Barazzone et al [27] as important elements in developing and maintaining a therapeutic relationship.

In terms of barriers identified in this trial, aspects of CBT for insomnia in general and program-specific technical issues in particular could have hindered the participants’ adherence to the Web-based program. For instance, many participants highlighted the intrinsic difficulty of sleep restriction as challenging. These challenges are present in face-to-face CBT for insomnia too. In their exploratory study on patients engaging in sleep restriction therapy for insomnia, Kyle et al [30] reported that one-third of the participants mentioned impaired driving ability due to sleep restriction, and several reported side effects (such as headache, nausea) during the first week of the restriction.

Some participants found the modules were too lengthy. The modules in SHUTi typically involve up to an hour of engagement, over a 9-week period [21]. Previous authors [31] have reported that CCBT programs are typically accessed for much briefer periods than the traditional therapy session duration of 50 minutes. As such, disaggregating the modules into shorter pieces might have helped engagement, which is key to an effective intervention [18].

### The Use of the CCBT-I in a Clinical Setting

Evidence from eHealth literature suggested that Web-based intervention might reduce stigma and allow more people seek help for mental health conditions [3]. In our sample, a small proportion of participants preferred the impersonal nature of using the Web over going through the cores with a clinician. One participant identified clinical care as a potential barrier due to the stigma of requiring psychiatric treatment. This might be due to one’s perception of mental health and cultural background, which influence their view on seeking psychiatric care.

In the qualitative data, the participants did not think the website helped reduce their contact with health professionals. Most participants reported that the regular clinical reviews facilitated their adherence to the Web-based program. This group said the clinical reviews provided them with a sense of accountability to complete the online tasks. They found that the reviews promoted their intrinsic motivation, and some added that the recommended clinical interventions, such as medications and mindfulness techniques, helped them to persist with the Web-based program. This may be why we did not identify some factors that emerged in nonclinical settings that were linked to persistence, such as a sense of “getting things done” and satisfaction at completing the program, as these may have been subsumed by the clinic.

The clinical management appeared to aid the CCBT-I by setting goals, planning treatment, and selecting intervention; that is, providing individualized treatment to the individual. This is essential in CBT, and indeed in any treatment. These elements would likely be more effectively achieved by incorporating a clinician into the Web-based intervention. On the other hand, the impersonal nature and constant availability of the Web-based program made attending the program more convenient for participants.

As Barazzone et al [27] suggested, CCBT-I programs encompass a substantial body of features essential to establishing a therapeutic relationship, but fewer features designed to develop and maintain a therapeutic alliance. Adjunctive clinical reviews could fill this void, by holding users through difficult phases of therapy, setbacks, and ruptures [32]. This may be a factor in the greater effectiveness of supported CCBT-I, as identified in the meta-analysis by Zachariae et al [14], and the clinical context of using unsupported CBT as an adjunctive treatment may lead to great benefits, although this comparison is yet to be tested.

Further, while most participants found the clinical intervention helpful, others found the clinical and computer program disjointed. This lack of coordination could be explained by the nature of the double-blind randomized trial. However, it highlights that in future commercially available CCBT-I programs need to have some facility to provide treating clinicians with information on where the participant is in the program, what interventions and messages are being delivered at that time, and any clinical progress. Otherwise, clinicians will be unable to integrate clinical intervention with the specific contents in the Web-based programs and potentially disengage from the programs. Clinicians in this study were therefore not aware of which intervention the participants received and were encouraged not to ask specific questions about the Web-based interventions in order to avoid unblinding.

### Limitations

There are several limitations to our study. Our results are unique to the targeted population (ie, men aged ≥50 years) and the analysis of this research team. Consideration should be given to the variance in results that may have been produced by focusing on a less-specific cohort. For example, studying women or a younger adult population could conceivably yield different findings. However, for the purposes of this study, this is of little consequence to the anticipated outcomes. The data analysis was also influenced by the perspective of the researchers. In this study, the primary author worked as a clinical psychiatrist. This position could have resulted in the development of clinically biased views of what influences adherence behavior in a clinical setting. This, however, may be different from the influences of adherence in a research or online forum. Given this, we attempted to be as objective as possible, but the analysis is likely to have been couched within these experiences.

The participants interviewed were people who volunteered to participate in a qualitative interview from a group that volunteered to participate in a trial. This group was therefore probably highly motivated to participate in research and may be different from those who do not wish to share their views. This is reflected in the reported emphasis of research as an important motivator by some interview participants.

Due to trial blinding constraints, we contacted participants over a period of months, some participants not until approximately 6 months after they had completed the intervention. This may have affected the ability of some participants to recall their intrinsic motivations for continuing to engage with the trial, and their recall is likely to have been influenced by their present situation. While some of the themes that emerged appear to be values based, and therefore quite static, it is likely that state variables such as time factors and frustration may not be as accurately reflected in these findings. Also, in the self-report data, the participants were not asked about what factors motivated their ongoing participation.

We conducted 4 of the 10 interviews over the telephone. While phone interviewing may have allowed respondents to feel relaxed and better able to disclose sensitive information, the absence of visual cues may have resulted in loss of contextual and nonverbal impressions on the interviewer, and may have inadvertently compromised rapport, probing, and interpretation of responses. By using a semistructured, standardized interview schedule, we attempted to consistently present interview questions in the same arrangement and verbal manner with all participants, while simultaneously encouraging elaboration of responses through directive provocation and unlimited time for responses. Further to this, the interviewer adhered to neutral responses (eg, “okay,” “go on”) to prevent leading the participant. Any environmental bias was minimized by the implementation of standard interview practice requiring a quiet, comfortable room in which to conduct the interview, and was moderated by any specific requests of the interviewee (eg, specific time of day).

Finally, we were only able to recruit participants who had completed the trial, although many had not necessarily completed all the modules; that is, they persisted with the trial but were not completely adherent to the intervention, or were withdrawn, preventing us from exploring the barriers and motivators in people who did not persist with the intervention. We were also not able to recruit anyone who completed the Web-based program but not the 6-month follow-up assessment. Of those who did not persist and did provide additional contact with the researchers, their reasons for dropping out of the trial tended to comprise of a lack of time to complete the program at present, and therefore an unwillingness to participate in a further interview. This, together with other limitations discussed above, might have contributed to an early saturation in our data collection after 10 interviews. This is a relatively small sample size in similar qualitative research. Future research would benefit from interviewing participants who have dropped out of Web-based interventions to determine their reasons for doing so.

### Conclusion

In terms of maintaining participants in Web-based programs, it may be useful to consider ways to maximize engagement and overcome the barriers that participants mentioned. The findings from this study indicated that the Web-based intervention benefitted those who were motivated, had a goal-directed personality, liked graphic interpretations, and were interested in improving their health holistically. The participants reported a benefit from the psychoeducation and behavioral reinforcement throughout the program in terms of engagement. Clinicians may capitalize on the motivation of this group of participants by providing regular feedback on the data provided and working on goals; for example, designing sleep restriction windows may enhance engagement and increase the likelihood of completion.

Other findings from this study suggested that care needs to be taken to ensure that messages and interventions given by the program and the clinician are coordinated if the use of the program is to be optimized. Future studies could further explore ways of integrating clinical input to enhance adherence and optimize treatment. For instance, Cavanagh [33] suggested that clinicians acknowledge both the strengths and limitations of CCBT-I programs, and offer them in the context of appropriate support that starts with discussion around the motivation, expectations, and preconceptions of the user regarding CCBT-I, addresses concerns, and promotes ongoing engagement with the program.

Clinicians may need to provide more additional support for those participants who are less motivated and goal directed. It would give these individuals realistic expectations and minimize anticipatory failure. Some participants mentioned online forums to promote social support and share knowledge. This may enhance a sense of community, and thus increase the likelihood of completing the program. Such forums have been successfully used in other programs [34]. Comparable with real-life clinical settings, there were participants in this study who had tried various interventions for their insomnia with little perceived benefit. This group might benefit from a more assertive and integrated approach in clinical and Web-based interventions.

Integrating automated eHealth interventions into routine clinical practice may improve outcomes; however, the programs and clinicians need to work in a synergistic fashion, sharing information and supporting the patient in their engagement with both aspects of treatment, if we are to realize the full potential of such approaches.

## Abbreviations

CBT: cognitive behavioral therapy
CCBT: computerized cognitive behavioral therapy
CCBT-I: computerized cognitive behavioral therapy for insomnia
DSM-5: Diagnostic and Statistical Manual of Mental Disorders
SOMNA: Sleep Or Mood Novel Adjunctive therapy
